# Age is the only predictor for upper gastrointestinal malignancy in Chinese patients with uncomplicated dyspepsia: a prospective investigation of endoscopic findings

**DOI:** 10.1186/s12876-021-01951-x

**Published:** 2021-11-23

**Authors:** Yu Huang, Qian Gui, Huiyi Li, Xiaohua Long, Xiao Liang, Hong Lu

**Affiliations:** 1 Division of Gastroenterology and Hepatology, Shanghai Institute of Digestive Disease; Key Laboratory of Gastroenterology & Hepatology, Ministry of Health; Ren Ji Hospital, School of Medicine, Shanghai Jiao Tong University, 145 Middle Shandong Road, Shanghai, 200001 China; 2 Department of Emergency; RenJi Hospital, School of Medicine, Shanghai Jiao Tong University, Shanghai, China

**Keywords:** Symptom, Endoscopy, Malignancy, Peptic ulcer

## Abstract

**Background:**

Dyspepsia is a common cause of physician visits. If and when endoscopy should be performed depend on the regions and the populations. This study aimed to identify the current risk factors predictive of upper gastrointestinal malignancy or peptic ulcer in China with high prevalence of gastric cancer.

**Methods:**

A questionnaire was conducted among consecutive outpatients undergoing their first esophagogastroduodenoscopy for dyspepsia. Symptoms other than alarm symptoms in this study were defined as uncomplicated dyspepsia.

**Results:**

4310 outpatients (mean age 44, median 42, range 14–86) were included in the final analyses. Significant pathology was found in 13.8% (595/4310) patients including peptic ulcer (12.3%) and upper gastrointestinal malignancy (1.5%). Age, male sex and alarm symptoms were significantly associated with malignancy. The age cut-off identified for upper gastrointestinal malignancy was 56 years among patients with uncomplicated dyspepsia, which was similar to the combined cutoff of age and gender.

**Conclusions:**

Age should be considered as the primary predictor for upper gastrointestinal malignancy in Chinese patients with uncomplicated dyspepsia. 56 could probably be the optimal age to identify those lesions in this population. Trial registration: Chictr.org (ChiCTR2000040775).

**Supplementary Information:**

The online version contains supplementary material available at 10.1186/s12876-021-01951-x.

## Background

Dyspepsia is a common complaint in China and although almost three-quarters of patients with dyspepsia do not have endoscopic findings [1, 2], peptic ulcers and gastric cancer are both frequent [3, 4]. The majority of gastric cancers worldwide occur in Asia with China being responsible for the majority of cases [4]. The status of upper gastrointestinal (GI) disease in China currently approximates that of early to mid-twentieth century in the United States or Europe with duodenal ulcer being common in the younger population and gastric ulcer and gastric cancer becoming more common as the population ages [5]. Gastroesophageal reflux is also common although the expression may differ from that commonly seen in the West [6]. For example, an endoscopic survey in Shanghai showed only 28.8% of patients with erosive esophagitis had typical symptoms [7].

In Japan, the population is encouraged to undergo upper gastrointestinal endoscopy as part of their regular physical examination starting at age 50 [8]. The role EGD in the evaluation of patients with uninvestigated dyspepsia varies depending on the background prevalence of peptic ulcer and gastric cancer in the population as well as factors such as age, sex, presence or absence of symptoms, family history, use of gastrotoxic or antisecretory drugs, etc [9]. The high prevalence of peptic ulcer and gastric cancer in China supports the use of endoscopy for evaluation of dyspepsia, its use for universal screening remains controversial in China and has not been adopted as standard policy. This contrasts with the use of endoscopy in Japan and South Korea, both high gastric cancer risk countries, where it is used to screen for early cancer has achieved good results [10].

The aim of this study was to investigate the prevalence of different upper gastrointestinal pathologies in a large Chinese population presenting to hospital because of upper gastrointestinal symptoms with the goal of identifying risk factors useful to predict major endoscopic findings in this population.

## Methods

### Study design and participants

This prospective observational study was carried out at the Digestive Endoscopy Center of Renji Hospital affiliated with Shanghai Jiao Tong University School of Medicine. From January to December 2017 consecutive adult outpatients presenting with upper gastrointestinal symptoms who had no prior investigations enrolled with no preference (dyspepsia, heartburn, alarm symptoms, etc.) were offered upper GI endoscopy. The exclusive criteria and questionnaire screening detail was shown in previous study [11]. Major symptom and symptom duration could be seen below for details. The EGD was performed by qualified gastroenterologists or endoscopic physicians, who were blinded to the included subjects. It is also routine practice at our endoscopic center to obtain biopsies for histological examination and rapid urease tests (RUT) for *H pylori* infection detection. All biopsy specimens were evaluated by experienced pathologists of the Department of Pathology of Renji Hospital. *H. pylori* infection was determined by one of positive outcome of rapid urease test (RUT) and histology or ^13^C urea breath test (UBT). Previous *H. pylori* infection was identified by the eradication history and its negative result of *H. pylori* in the study.

### Definitions

The Rome IV diagnostic criteria [12] for dyspepsia were used and included upper abdominal symptoms including postprandial fullness, early satiety, epigastric pain or epigastric burning. Reflux symptoms were defined as heartburn, regurgitation, or retrosternal pain according to the Montreal Consensus [13]. Alarm symptoms considered in this study included melena, vomiting, anemia, weight loss (more than 10% weight loss in six month), hematemesis or vomiting brown liquid or dysphagia. Other symptoms scored included belching, poor appetite, retrosternal discomfort, abdominal discomfort, nausea, hiccup, bad breath and pharyngeal symptoms. Symptoms other than alarm symptoms in this study were defined as uncomplicated dyspepsia. The principle for ranking of multiple symptoms was alarm symptoms first and followed by the most troublesome symptom. Based on clinical practice, the symptoms in this study were divided into the following 5 groups based on symptoms duration (x): < 1 week, 1 week ≤ x < 1 month, 1 month ≤ x < 3 months, 3 months ≤ x < 6 months, ≥ 6 months. Endoscopic findings were defined in previous study [11].

### Statistical analysis

Statistical analysis of the data was performed with SPSS 18.0 and MedCalc Version 19.2.0 for Windows (Statistical Product and Service Solutions). According to endoscopic findings, patients were divided into 3 groups: malignancy, peptic ulcer and non-major lesions. The influence factors between the different subgroups were analyzed by univariate logistic correlation analysis and multivariate logistic stepwise regression and their odd ratios (OR), 95% confidence intervals and p values were calculated. A *p* value < 0.05 was considered statistically significant. The diagnostic accuracy of related factors was evaluated with a receiver operating characteristic (ROC) curve and area under curve (AUC). The curve represents the relationship between sensitivity and specificity for the prediction of major lesions. The cutoff value of age for malignancy was determined by Youden’s index.

## Results

### Demographic and clinical data of patients

A total of 4624 patients completed the survey questionnaire and 4310 (93.2%) underwent EGD. Patient demographic data are shown in Additional file 1: Table S1. There were no statistically significant differences in gender (*p* = 0.342) or age (*p* = 0.194) between study population and the population lost to follow-up. RUT was not performed in 19 patients or/and *H. pylori* histology was not done because a malignant lesion obstruction prevented endoscopic entry or they were taking anticoagulant drugs. In those subjects active *H. pylori* infection was determined by ^13^C urea breath test or the positive serum antibody without eradication history.

EGDs were normal in 75.1%. Major lesions were found in 13.8% (595 patients) (Table 1) including 12.3% (529 patients) with peptic ulcers, 350 duodenal ulcers (8.1%), 125 gastric ulcers (2.9%), and 54 gastric and duodenal ulcers (1.3%). There were 66 malignancies (1.5%) including 51 gastric cancers (1.2%, 7 early, 44 advanced) and 15 esophageal cancers (0.3%, 13 squamous, 2 adenocarcinoma).

**Table 1:** Endoscopic findings (n = 4310)

Normal appearance	3235 (75.1%)
Reflux esophagitis	486* (11.3%)
Peptic ulcer	529 (12.3%)
Gastric ulcer	125 (2.9%)
Duodenal ulcer	350 (8.1%)
Compound ulcer	54 (1.3%)
Malignancy	66 (1.5%)
Gastric cancer	51 (1.2%)
Esophageal cancer	15 (0.3%)
Other (submucosal masses, gastric adenoma, neuroendocrine tumor, etc.)	59 (1.4%)

*Esophagitis combined with peptic ulcer: 64 cases; esophagitis combined with gastric cancer: 1 case

The prevalence of endoscopic findings in relation to symptoms is shown in Additional file 1: Table S2. Malignancies first appeared in the 30–40 year age group and the prevalence increased rapidly with age (Additional file 1: Table S3). A total of 1475 patients (34.2%) had active *H. pylori* infections including 72.4% of those with peptic ulcers and 24.2% with malignancy (esophagus cancer 26.7%, gastric cancer 23.5%). Erosive esophagitis was present in 11.3%. Except for peptic ulcer, the prevalence of active *H. pylori* infection was similar in all groups: dyspeptic symptoms (29.8%), reflux symptoms (25.6%), alarm symptoms (26.8%) and other symptoms (27.3%) (Additional file 1: Figure S1).

### Factors associated with the major lesions

Age, male sex and alarm symptoms were the risk factors significantly associated with malignancy (Table 2). Age, male sex, smoking history, alarm symptoms, symptom duration of < 1 week and active *H. pylori* infection were significantly related to peptic ulcer (Table 3).

**Table 2:** Univariate logistic analysis and multivariate logistic regression analysis of related factors of malignancy

Factors	Univariate		Multivariate	
	OR (95% CI)	*p*	OR (95% CI)*	*p*
Age, per 1 year	1.12 (1.09–1.15)	≤ 0.001	1.12 (1.09–1.14)	≤ 0.001
Gender, male	4.55 (2.58–8.01)	≤ 0.001	4.08 (2.24–7.43)	≤ 0.001
Smoking history	3.27 (1.54–6.95)	0.002	1.36 (0.59–3.12)	0.466
Drinking history	1.80 (0.65–5.01)	0.262		
Family history	0.61 (0.08–4.41)	0.621		
Family income per capita		0.593		
< 3000 yuan/month	1.62 (0.64–4.07)	0.307		
3000–10,000 yuan/month	1	–		
> 10,000 yuan/month	≤ 0.001	0.996		
Symptom		≤ 0.001		
Dyspeptic symptoms	1.47 (0.57–3.75)	0.423	1.74 (0.67–4.54)	0.256
Reflux symptoms	0.87 (0.21–3.67)	0.852	1.01 (0.24–4.37)	0.986
Alarm symptoms	9.88 (3.69–26.50)	≤ 0.001	8.24 (2.90–23.46)	≤ 0.001
Other symptoms	1	–	1	–
Symptom duration		0.651		
x < 1 week	1.47 (0.61–3.51)	0.391		
1 week ≤ x < 1 month	1.02 (0.50–2.06)	0.967		
1 month ≤ x < 3 months	1.40 (0.74–2.63)	0.302		
3 months ≤ x < 6 months	1.68 (0.70–4.03)	0.245		
≥ 6 months	1	–		
Current *H. pylori* infection	0.61 (0.35–1.08)	0.088		
Current and previous *H. pylori* infection	0.58 (0.34–1.0)	0.049	0.65 (0.36–1.16)	0.142

*Adjusted by age, gender, smoking history, symptom, symptom duration, current and previous *H. pylori* infection

**Table 3:** Univariate logistic analysis and multivariate logistic regression analysis of related factors of peptic ulcer

Factors	Univariate		Multivariate	
	OR (95% CI)	*p*	OR (95% CI)*	*p*
Age, per 1 year	1.01 (1.00–1.01)	0.020	1.01 (1.00–1.02)	0.004
Gender, male	2.77 (2.29–3.34)	≤ 0.001	2.40 (1.95–2.95)	≤ 0.001
Smoking history	4.23 (3.07–5.84)	≤ 0.001	2.13 (1.40–3.23)	≤ 0.001
Drinking history	2.33 (1.58–3.42)	≤ 0.001	0.86 (0.52–1.41)	0.536
Family history	0.91 (0.50–1.67)	0.762		
NSAIDs and antithrombotic agents	1.22 (0.60–2.48)	0.588		
Family income per capita		0.049		
< 3000 yuan/month	1.50 (1.03–2.19)	0.035	1.47 (0.97–2.23)	0.072
3000–10,000 yuan/month	1	-	1	-
> 10,000 yuan/month	0.68 (0.35–1.30)	0.243	0.67 (0.33–1.33)	0.250
Symptom		0.004		
Dyspeptic symptoms	1.39 (1.03–1.87)	0.031	1.30 (0.94–1.78)	0.110
Reflux symptoms	1.43 (0.96–2.14)	0.081	1.40 (0.91–2.16)	0.127
Alarm symptoms	2.16 (1.43–3.27)	≤ 0.001	1.84 (1.16–2.90)	0.009
Other symptoms	1	–	1	–
Symptom duration		0.012		
x < 1 week	1.75 (1.28–2.39)	0.001	1.75 (1.24–2.49)	0.002
1 week ≤ x < 1 month	1.26 (0.98–1.61)	0.071	1.28 (0.98–1.67)	0.072
1 month ≤ x < 3 months	1.13 (0.88–1.44)	0.337	1.09 (0.84–1.41)	0.539
3 months ≤ x < 6 months	1.16 (0.80–1.67)	0.440	1.18 (0.79–1.76)	0.411
≥ 6 months	1	–	1	–
Current *H. pylori* infection	6.46 (5.27–7.92)	≤ 0.001	6.37 (5.17–7.85)	≤ 0.001

*Adjusted by age, gender, smoking history, drinking history, family income per capita, symptom, symptom duration and *H. pylori*

**Fig. 1:**
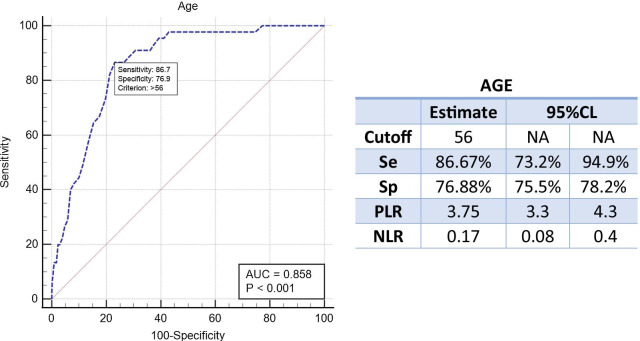
Age indication for malignancy in patients with uncomplicated dyspepsia

The subgroup of 4037 patients with uncomplicated dyspepsia has 45 malignancies (68.2%, 12 females; 33 males). Four patients (6.1%) were aged less than 50 years. Age cutoffs derived from the ROC curve of Youden’s index were 56 years for males and 57 years for females, with 28 of 33 and 11 of 12 cancers detected over these thresholds, respectively. Table 4 shows the effect of combination of age and gender on the predictive capability of malignancy in patients with uncomplicated dyspepsia. Shifting the age cut-off to 56 for males and to 57 for females compared to the recommended age of 50 in Japan showed a numerical increase for the positive likelihood ratio obviously and also improved the sensitivity and specificity relatively. The age cut-off identified for upper gastrointestinal malignancy was 56 years among patients with uncomplicated dyspepsia (Fig. 1), which is similar to combined cut-off of age and gender.

**Table 4:** Effect of combination of age and Gender on the predictive capability of malignancy in patients with uncomplicated dyspepsia (n = 4037)

	Age		Gender		Gender for malignancy			
					F	F	M	M
	< 50	> 50	Female	Male	< 57	> 57	< 56	> 56
Total n. patients	2553	1484	2397	1640	1857	540	1266	374
Malignancy (n, 45)	4	41	12	33	1	11	5	28
OR	1.12		4.40		1.12		1.12	
[95% CI]	[1.09, 1.15]		[2.24, 8.62]		[1.06, 1.18]		[1.08, 1.16]	
AUC	0.775		0.665		0.841		0.871	
[95% CI]	[0.721, 0.829]		[0.651, 0.680]		[0.826, 0.856]		[0.854, 0.887]	
Sensitivity	91.11%		73.33%		91.67%		84.85%	
[95% CI]	[78.8, 97.5]		[58.1, 85.4]		[61.5, 99.8]		[68.1, 94.9]	
Specificity	65.53%		59.74%		77.82%		78.47%	
[95% CI]	[64.0, 67.0]		[58.2, 61.3]		[76.1, 79.5]		[76.4—80.5]	
LR+	2.64		1.82		4.13		3.94	
[95% CI]	[2.4, 2.9]		[1.5, 2.2]		[3.4, 5.0]		[3.3—4.7]	
LR-	0.14		0.45		0.11		0.19	
[95% CI]	[0.05, 0.3]		[0.3, 0.7]		[0.02, 0.7]		0.09—0.4	

*OR* odd ratios, *AUC* area under curve, *95% CI* confidence interval, *LR* likelihood

## Discussion

In this prospective study of 4310 consecutive patients undergoing upper gastrointestinal endoscopy for evaluation of upper gastrointestinal symptoms, 75.1% had a normal EGD; 13.8% had major lesions (peptic ulcer disease 12.3%, gastric cancer 1.2%, and esophageal cancer 0.3%). These results are consistent with another study from China showing that as least a quarter of patients undergoing EGD had significant endoscopic findings [14]. These findings contrast with those of western countries where reflux esophagitis and endoscopic suspected esophageal metaplasia (ESEM) are currently the predominant findings. The high prevalence of *H. pylori* and its related diseases, peptic ulcer and malignancy, remains high in China [14] such that in China, in contrast to many western countries, screening for peptic ulcer and malignancy has proven to be cost effective [15, 16].

The prevalence of peptic ulcer (12.3%) is slightly below the range of the endoscopic case series reported in China (13.7–22.5%) [17, 18], probably due to the relatively low prevalence of *H. pylori* infection (34.2% in the total study sample). This prospective study revealed a strong positive association between active *H. pylori* infection and peptic ulcer. The majority of individuals with peptic ulcer (72.4%) in the study were found to be *H. pylori* positive. Age, male sex, smoking history, alarm symptoms and symptom duration of < 1 week were also independent predictors for developing peptic ulcer. NSAID use, known to be associated with gastrointestinal injury, including erosions, ulceration and hemorrhage, was not identified as a risk factor for the development of symptomatic peptic ulcer in this study.

The diagnostic value of any symptom or set of common symptoms is generally related to the prevalence of the disease being sought in that population. As such, gastric cancer is expected to be a rare endoscopic finding for evaluation of dyspepsia in the United States [19] and to become increasingly common in proportion to the incidence of gastric cancer in the population [20]. In China *H. pylori* infection and its related diseases are common as confirmed in this study 12.3% and 1.2% of dyspeptic patients were diagnosed with peptic ulcer and gastric cancer, respectively. Our study also confirmed that most patients presenting with dyspepsia have a normal endoscopy which is reflected in the lack of a significant association between the presence of dyspepsia and finding a peptic ulcer or gastric cancer.

The value of alarm symptoms remains controversial, particularly in low cancer incidence countries where alarm symptoms, with the possible exception of dysphagia and weight loss, have little or no predictive value [21, 22]. Even in areas with a high rate of esophago-gastric cancer only 7.7% of patients with alarm symptoms had an upper gastrointestinal malignancy. For the 31.8% of patients with malignancy in this study the presence of alarm symptoms had an OR of 8.54.

Although upper GI endoscopy is an important means of diagnosing upper GI diseases, its cost effectiveness as a screening modality depends on the costs and expected findings in the candidate population. In western countries, the most common clinical diseases associated with uninvestigated dyspepsia are gastroesophageal reflux disease (40%), non-ulcer dyspepsia (40%) and peptic ulcer (13%) [23]. In those populations gastric cancer is rare and endoscopic screening of patients with uninvestigated dyspepsia is generally not recommended until an age of 60 [24]. All people more than 50 years old in Japan were required to undergo endoscopy to rule out organic disease [8]. Based on the results of the present study, in the subgroup of 45 patients with uncomplicated dyspepsia who had malignancy at EGD, the Japan’s recommend age cut-off of 50 years demonstrated a lower positive likelihood ratio for predicting malignancy. Better clinical predictors for upper GI malignancy need to be identified. Given the high prevalence of dyspepsia, the immediately endoscopic investigation in the cut-off age is not a trivial exercise. Thus, even a variation of a few years in the cut-off age would change management strategy in thousands of people especially in China with a large population basis. Our study suggests the age cut-offs identified for upper GI malignancy was 56 years for males and 57 years for females in patients with uncomplicated dyspepsia, which is similar to the age cut-off alone (56 years). However, a prospective multicenter study in Italy showed that the age cut-offs identified for malignancy with uncomplicated dyspepsia were 35 years for males and 56 years for females [25].

Compared to the previous study, we had some characteristics. Patients with the first EGD were selected as the target population, so the indications of endoscopy were diverse and comprehensive. Length of symptom and *H. pylori* infection were also included, which were rarely mentioned in previous studies. Moreover, this study originally find out that the age cut-off for malignancy is 56 year which isn’t basically affected by gender among patients with uncomplicated dyspepsia in China with high background prevalence of *H. pylori* infection and upper GI malignancy. The limitation of this study was that this was a single-center observational study and 314 patients did not finish EGD.

## Conclusions

Age should be considered as the primary predictor for malignancy in Chinese with uncomplicated dyspepsia regardless of the gender. Our data strongly suggest that 56 could probably be the optimal age to identify those lesions in this population.

## Supplementary Information

**Additional file 1**. **Supplemental Table 1.** Basic characteristics of patients studied (n=4310). **Supplemental Table 2.** The prevalence of endoscopic findings in various symptoms. **Supplemental Table 3.** The prevalence of gastric pathology in different ages. **Supplemental Figure 1.** The prevalence of *H. pylori* infection in patients with various symptoms.

## Data Availability

The datasets generated and analysed during the current study are not publicly available due privacy issues, but are available from the corresponding author on reasonable request.

## Abbreviations

EGD: Esophagogastroduodenoscopy
GI: Gastrointestinal
RUT: Rapid urease tests
UBT: Urea breath test
OR: Odd ratios
ROC: Receiver operating characteristic
AUC: Area under curve
