# Supplementary material for: Age is the only predictor for upper gastrointestinal malignancy in Chinese patients with uncomplicated dyspepsia: a prospective investigation of endoscopic findings
Source: BMC Gastroenterol. 2021 Nov 23;21:441. doi: 10.1186/s12876-021-01951-x (PMC8609854; doi:10.1186/s12876-021-01951-x)
Supplement: Supplementary file 1 — Additional file 1. Supplemental Table 1. Basic characteristics of patients studied (n=4310). Supplemental Table 2. The prevalence of endoscopic findings in various symptoms. Supplemental Table 3. The prevalence of gastric pathology in different ages. Supplemental Figure 1. The prevalence of H. pylori infection in patients with various symptoms. [file 12876_2021_1951_MOESM1_ESM.docx]

Supplemental Table 1. Basic characteristics of patients studied (n=4310)

| Age (mean±SD) | 44.1±14.2 |
| --- | --- |
| Gender |  |
| Male | 1779 (41.3%) |
| Female | 2531 (58.7%) |
| Family income per capita |  |
| ＜3000 yuan/ month | 204 (4.7%) |
| 3000-10000 yuan/month | 3989 (92.6%) |
| ＞10000 yuan/ month | 117 (2.7%) |
| Family history |  |
| Yes | 106 (2.5%) |
| No | 4204 (97.5%) |
| Smoking history |  |
| Yes | 180 (4.2%) |
| No | 4130 (95.8%) |
| Drinking history |  |
| Yes | 151 (3.5%) |
| No | 4159 (96.5%) |
| NSAIDs and antithrombotic agents |  |
| Yes | 62 (1.4%) |
| No | 4248 (98.6%) |
| Symptom |  |
| Dyspeptic symptoms | 3028 (70.3%） |
| Reflux symptoms | 411 (9.5%） |
| Alarm symptoms | 273 (6.3%） |
| Other symptoms | 598 (13.9%） |
| Symptom duration |  |
| x < 1 week | 375（8.7%） |
| 1 week ≤ x < 1 month | 1000（23.2%） |
| 1 month ≤ x < 3 months | 1124（26.1%） |
| 3 months ≤ x < 6 months | 328（7.6%） |
| ≥ 6months | 1483（34.4%） |
| Active *H. pylori* infection |  |
| Yes | 1475 (34.2%) |
| No | 2835 (65.8%) |

Supplemental Table 2. The prevalence of endoscopic findings in various symptoms

| n (%) | Normal appearance | Reflux esophagitis | Peptic ulcer | Malignancy | Other |
| --- | --- | --- | --- | --- | --- |
| Dyspeptic symptoms  (n=3028) | 2328 (76.9) | 290 (9.6) | 373 (12.3) | 37 (1.2) | 41 (1.4) |
| Reflux symptoms  (n=411) | 260 (63.3) | 106 (25.8) | 52 (12.7) | 3 (0.7) | 3 (0.7) |
| Alarm symptoms  (n=273) | 171 (62.6) | 34 (12.5) | 49 (17.9) | 21 (7.7) | 4 (1.5) |
| Other symptoms  (n=598) | 476 (79.6) | 56 (9.4) | 55 (9.2) | 5 (0.8) | 11 (1.8) |

Supplemental Table 3. The prevalence of gastric pathology in different ages

| Age group | ~30  (n=855) | 31-40  (n=1163) | 41-50  (n=752) | 51-60  (n=836) | 61-70  (n=585) | >70  (n=119) |
| --- | --- | --- | --- | --- | --- | --- |
| Malignancy (n, %) | 0  (0%) | 1  (0.1%) | 3  (0.4%) | 21  (2.5%) | 29  (5.0%) | 12  (10.1%) |
| Peptic ulcer  (n, %) | 96  (11.2%) | 125 (10.7%) | 103 (13.7%) | 108 (12.9%) | 77  (13.2%) | 20  (16.8%) |
| Erosive GERD (n, %) | 72  (8.4%) | 133  (11.4) | 89  (11.8%) | 97  (11.6) | 75  (12.8%) | 19  (16.0%) |


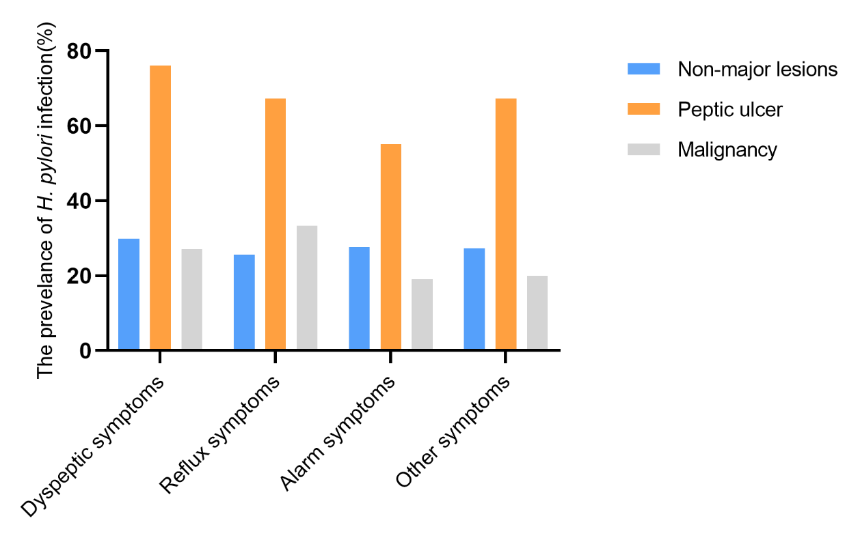


Supplemental Figure 1. The prevalence of *H. pylori* infection in patients with various symptoms
